# Synovial Fluid Volume at the Time of Arthroscopic Rotator Cuff Repair Correlates With Tear Size

**DOI:** 10.7759/cureus.9224

**Published:** 2020-07-16

**Authors:** Michael Stone, Grant Jamgochian, Ocean Thakar, Manan S Patel, Joseph A Abboud

**Affiliations:** 1 Shoulder and Elbow Surgery, Cedars-Sinai Medical Center, Los Angeles, USA; 2 Shoulder and Elbow Surgery, Rothman Institute at Thomas Jefferson University, Philadelphia, USA; 3 Orthopaedics, MedStar Union Memorial Hospital, Baltimore, USA; 4 Shoulder and Elbow Surgery, Rothman Orthopaedic Institute, Philadelphia, USA

**Keywords:** synovial fluid, rotator cuff tear, rotator cuff repair, inflammation, arthroscopy

## Abstract

Background

Inflammatory biomarkers are increased in the synovium and tendon of rotator cuff tears. Several studies demonstrate an associated increase in these markers and size of the tear, with implications of chondral destruction leading to rotator cuff tear arthropathy and glenohumeral arthritis.

Methods

This is a prospective study of 105 patients undergoing arthroscopic rotator cuff repair in which intra-articular synovial fluid was aspirated just prior to arthroscopy. Adult patients with a partial or full-thickness rotator cuff tear undergoing arthroscopic repair were included, and those with inflammatory arthritis, active infection, open cuff repair, intraoperative findings of osteoarthritis, or those undergoing revision cuff repair were excluded.

Results

The average patient age was 58 years (range 33-74 years), with 59 (56.2%) males. The mean aspirate volume of partial tears was 0.76 ± 0.43 mL, small tears 1.46 ± 1.88 mL, medium tears 3.04 ± 2.21 mL, and large tears 6.60 ± 3.23 mL. Full-thickness versus partial tears had significantly more synovial fluid (3.64 vs. 0.76 mL, respectively, p < 0.0001). An aspiration volume of 1.5 mL or greater resulted in 91.3% specificity and 96.8% positive predictive value for a full-thickness tear. Smoking (p = 0.017), tear size (p < 0.0001), and tears of the infraspinatus (p = 0.048) were significantly correlated with synovial fluid volume. Age, body mass index, chronicity of tear, sex, subscapularis involvement, supraspinatus involvement, and teres minor involvement had no association to synovial fluid volume.

Conclusion

Preoperative aspiration of the glenohumeral joint to identify the volume of synovial fluid can aid to identify full-thickness rotator cuff tears, and increased fluid volume should alert the clinician of a large tear.

## Introduction

It has been reported that in some instances rotator cuff pathology can rapidly accelerate the destruction of the shoulder joint [1-4]. Due to the role synovial fluid plays in nutrition and in the excretion of the metabolic components of cartilage in the shoulder, characterization of its content has been proposed as a viable medium to directly reflect joint pathology [4]. Previous studies investigating synovial fluid content have demonstrated that greater levels of synovial inflammation, defined as the upregulation of inflammatory biomarkers (i.e. matrix metalloproteinases, MMP-1 and MMP-3, and cartilage-degrading enzymes produced by synovial lining and chondrocytes) and angiogenesis, correlate with rotator cuff tear size [4]. Shindle et al. demonstrated that increased synovial inflammation and tissue degeneration were associated with greater tear sizes of the supraspinatus tendon [5]. Furthermore, Abrams et al. reported similar results in which patients with full-thickness rotator cuff tears had greater levels of synovial inflammation compared to patients without tears [6]. Shih et al. found a significant correlation between elevated IL-1ß (interleukin), levels in the synovial fluid of patients with rotator cuff tears and pain, and lower functional outcome scores [7]. To date, however, no study has attempted to provide a quantitative analysis to correlate the volume of synovial fluid aspirated at the time of rotator cuff repair to the size of rotator cuff tear.

MRI and other forms of diagnostic imaging do not always accurately depict the size and number of tendons involved in rotator cuff tears [8]. Frequently, intraoperative findings include a tear much larger than determined on MRI, or the lack of a tear at all. Finding alternative methods to diagnose and define the characteristics of rotator cuff tears will help the surgeon reaffirm expected preoperative findings at the time of arthroscopy as well as potentially give the patient more information about their healing potential and quality of life after surgery. The purpose of this study was to analyze the volume of synovial fluid aspirated in patients with a partial or full-thickness rotator cuff tear at the time of arthroscopic surgery and correlate to tear size. 

## Materials and methods

This is a prospective study that involved patients undergoing arthroscopic rotator cuff repair surgery. Inclusion criteria consisted of patients 18 years of age or older with a partial or full-thickness rotator cuff tear undergoing arthroscopic repair. Exclusion criteria consisted of patients younger than 18 years of age, presence of osteoarthritis, inflammatory arthritis, active infection, open cuff repair, or those undergoing revision cuff repair. Patients were first screened in the clinic to determine the characteristics of their rotator cuff tear by physical examination and MRI. They were enrolled into the study once they had a confirmed high-grade partial or full-thickness rotator cuff tear.

During the procedure, an initial standard posterior portal is established, and a trocar is placed within this portal. Any immediate fluid released from the joint is collected in a specimen cup. A dry arthroscope is then placed into the glenohumeral joint. A spinal needle is placed through the rotator interval and connected to a 10 cc syringe to collect any additional joint fluid under direct visualization. All synovial fluid is aspirated and confirmed by looking at the axillary recess. The amount of fluid was then measured by the surgeon, confirmed by a second research assistant, and recorded. Rotator cuff tear size was assessed intraoperatively as described by Post et al., measuring the tear in its longest diameter, with a small tear defined as <1 cm, medium tear as <3 cm, large tear as <5 cm, and a massive tear as 5 cm or greater [9].

Patient demographics, size of tear, mechanism of injury, chronicity of the tear, comorbidities, tendon involvement, range of motion, and the amount of synovial fluid aspirated were all documented and analyzed (Table 1).

**Table 1:** Patient demographics and tear characteristics M, male; F, female; L, left; R, right; BMI, body mass index; ROM, range of motion; SD, standard deviation

Variable	Mean ± SD (range) or no. (%) (n=105)
Age	58 ± 9 (range 33-74)
Sex	M = 59 (56.2%), F = 46 (43.8%)
Side	L = 35 (33.3%), R = 70 (66.7%)
Height (in)	67.18 ± 4.59 (range 54-77)
Weight (lbs)	189.90 ± 41.77 (range 95-305)
BMI	29.33 ± 5.48 (range 18.6-43.5)
Smoker	14 (13.3%)
Tear size	
Partial	23 (21.9%)
Small	26 (24.8%)
Medium	27 (25.7%)
Large	24 (22.9%)
Massive	5 (4.8%)
Tear chronicity	
Acute	15 (14.9%)
Chronic	53 (52.5%)
Acute on chronic	33 (32.7%)
Anchors	2.14 ± 1.30 (range 1-7)
Tendon involvement	
Supraspinatus	91 (86.7%)
Infraspinatus	28 (26.7%)
Subscapularis	31 (29.5%)
Teres Minor	1 (0.9%)
ROM (°)	
Forward elevation	124.90 ± 40.96 (range 20-170)
External rotation	41.91 ± 19.06 (range 0-90)

Statistics

Summary statistics, including means and standard deviations, were calculated. The Shapiro-Wilk test was used to determine normality of data. Comparison of means with multiple variables was performed using the Kruskal-Wallis test for data with nonparametric distribution. The analysis of variance (ANOVA) test was used for comparisons of means for data with parametric distributions. The Mann-Whitney U test was used for comparison of means between groups with nonparametric data. Univariate logistic regression for pairwise comparisons was performed using the Bonferroni correction. A multiple regression model was used to analyze covariates with fluid aspirate. All statistics were performed using the Stata software (StataCorp, College Station, TX, USA). Signiﬁcance was set as p < 0.05.

## Results

A total of 125 patients with partial or full-thickness rotator cuff tears were included in the initial review. Out of 125 patients, seven were excluded for shoulder osteoarthritis confirmed on arthroscopy, four were undergoing revision cuff repair, two had inflammatory arthritis (lupus or rheumatoid arthritis on active treatment), and seven had inadequate data, leaving a total of 105 patients for final analysis after exclusion. There were 23 partial-thickness tears (21.9%), 26 small tears (24.8%), 27 medium tears (25.7%), 24 large tears (22.9%), and five massive tears (4.8%).

**Figure 1:**
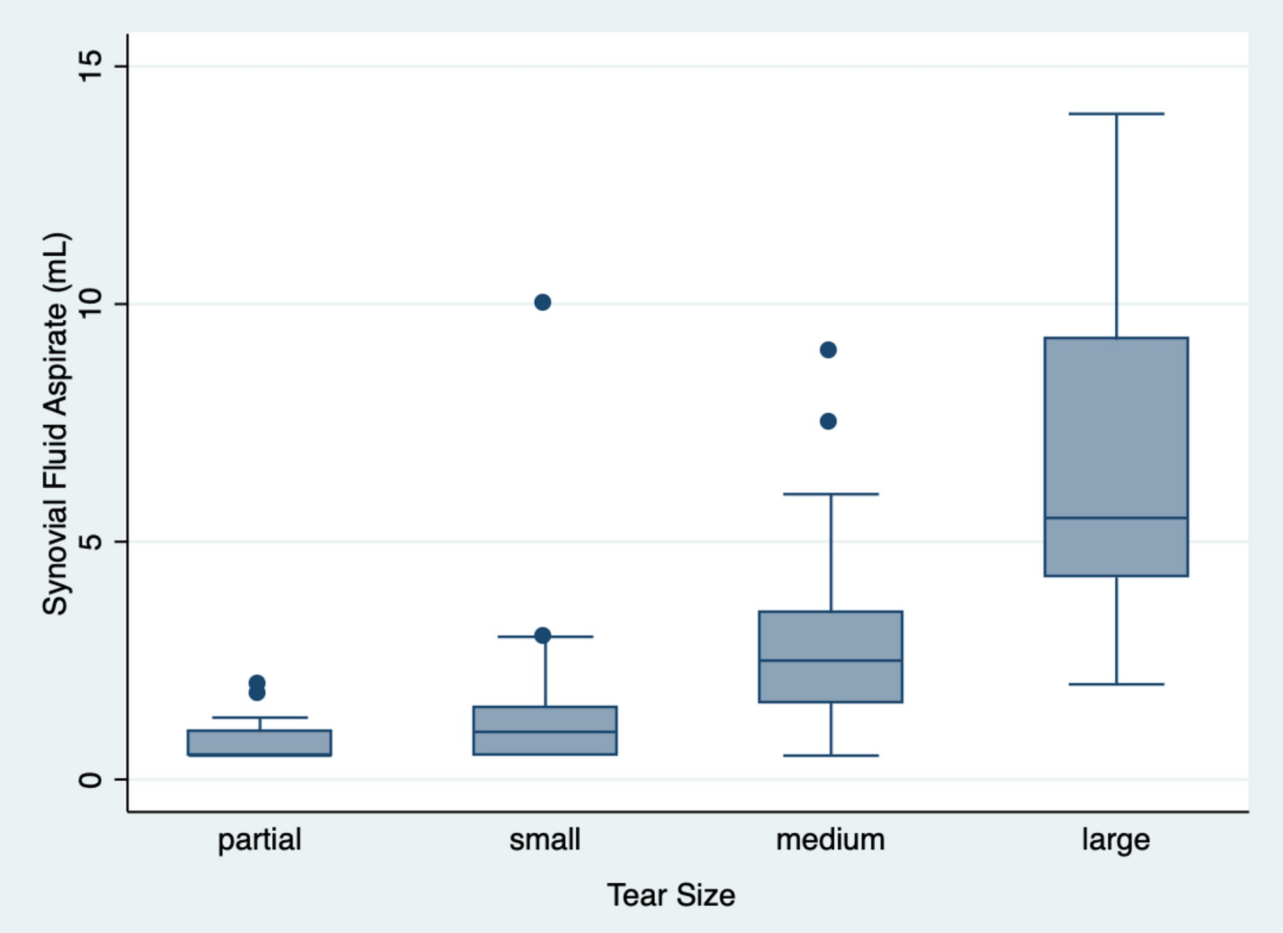
Synovial fluid aspirate volume based on tear size

Partial-thickness tears had a mean aspirate volume of 0.76 ± 0.43 mL, small tears had a mean aspirate volume of 1.46 ± 1.88 mL, medium tears had a mean aspirate volume of 3.04 ± 2.21 mL, and large tears had a mean aspirate volume of 6.60 ± 3.23 mL (Figure 1). There was a statistically significant difference in synovial fluid volume based on tear size (p < 0.0001). Chronicity of tear showed a trend toward a statistically significant difference in synovial fluid aspirate volume (p = 0.057) (Table 2).

**Table 2:** Mean synovial fluid aspirated based on tear size and chronicity *Indicates statistically significant difference

Variable	Mean (mL)	P value
Tear size		<0.0001*
Partial	0.76 ± 0.43	
Small	1.46 ± 1.88	
Medium	3.04 ± 2.21	
Large	6.60 ± 3.23	
Tear chronicity		0.057
Acute	5.25 ± 3.18	
Chronic	2.33 ± 2.54	
Acute on chronic	3.40 ± 3.51	

Pairwise comparisons of mean fluid aspirated between tear sizes showed statistically significant differences when comparing medium vs partial (3.04 vs 0.76 mL, p = 0.004), large vs partial (6.60 vs 0.76 mL, p < 0.0001), large vs small (6.60 vs 1.46 mL, p < 0.0001), and large vs medium sized tears (6.60 vs 3.04 mL, p < 0.0001). A comparison of small vs partial and medium vs small tears did not show a statistically significant difference between groups (Table 3). A comparison of partial-thickness tears and full-thickness tears of any size showed a statistically significant difference in mean synovial fluid aspirate with full-thickness tears having a higher volume (3.64 vs 0.76 mL, p < 0.0001). We did not have an adequate number of massive tears to perform a meaningful statistical analysis for these tears.

**Table 3:** Pairwise comparisons of fluid aspirate based on tear size *Indicates a statistically significant difference

Pairwise comparisons	Mean difference in synovial fluid aspirate (mL)	P value
Small vs partial	0.71	1.000
Medium vs partial	2.28	0.004*
Large vs partial	5.85	<0.0001*
Medium vs small	1.58	0.100
Large vs small	5.14	<0.0001*
Large vs medium	3.56	<0.0001*
Partial vs full-thickness tears (all)	2.88	<0.0001*

In an attempt to determine useful cutoff values for diagnostic accuracy of aspirate volume, a sensitivity, specificity, positive predictive value (PPV), and negative predictive value (NPV) were calculated (Table 4). An aspirate volume of ≥2.5 mL had 100% specificity and PPV for a full-thickness rotator cuff tear. A cutoff value of ≥1.5 mL resulted in 91.3% specificity and 96.8% PPV for a full-thickness tear. The accuracy of correctly diagnosing a full-thickness tear dropped significantly when below this value.

**Table 4:** Sensitivity, specificity, positive predictive value, and negative predictive values for a full-thickness cuff tear PPV, positive predictive value; NPV, negative predictive value

Fluid aspirate (mL)	Sensitivity	Specificity	PPV	NPV
1	82.9%	69.6%	90.6%	53.3%
1.5	73.1%	91.3%	96.8%	48.8%
2	61.0%	95.7%	98.0%	40.7%
2.5	51.2%	100%	100%	36.5%

A multiple logistic regression model was used to identify variables correlating with fluid aspiration. Smoking (p = 0.017), tear size (p < 0.0001), and tears of the infraspinatus (p = 0.048) had a statistically significant correlation with synovial fluid aspiration volume. Age, body mass index, chronicity of tear, sex, subscapularis involvement, supraspinatus involvement, and teres minor involvement had no statistically significant association to fluid aspiration volume.

## Discussion

The results of the current study show that synovial fluid volume present on aspiration prior to arthroscopic rotator cuff repair correlates with tear size. Pairwise comparisons between tear sizes showed an overall ability to differentiate between tear size and amount of fluid aspirated. The results represent an ability to differentiate between smaller tears compared to bigger tears, but difficulty in differentiating between tears of similar size. This is the first study, to our knowledge, to differentiate cuff tear size based on preoperative synovial fluid aspiration.

The correlation between synovial inflammation and rotator cuff disease has been well established in the literature. Based on the work of Shindle et al., there are several proinflammatory cytokines expressed in both the synovium and torn supraspinatus tendon itself in full-thickness tears compared to partial-thickness tears [5]. The authors found significantly elevated levels of IL-1β, IL-6, tumor necrosis factor (TNF-α), inducible nitric oxide synthase (iNOS), cyclooxygenase-2 (COX-2), and vascular endothelial growth factor (VEGF) in synovial tissue of shoulders with full-thickness rotator cuff tears. This inflammatory cascade is hypothesized to be implicated as the initial step in cartilage degeneration in relation to cuff tear arthropathy. Our study identified a significant correlation to size of tear and volume of synovial fluid aspirated, suggesting that tear size may be directly related to inflammation. Abrams et al. found significantly increased gene expression of inflammatory mediators in full-thickness rotator cuff tears, indicating a significant increase in synovial inflammation. These markers included common-leukocyte antigen, CD31, CD45, and CD68, along with inflammatory mediators MMP-3 and IL-6, compared to control [6]. However, we did not look specifically at the cytokine profile of the synovial aspirate, and thus such a conclusion cannot be drawn at present. 

After rotator cuff tears, there are three stages for healing of the rotator cuff: (1) inflammatory phase, (2) repair phase, and (3) remodeling phase [10]. In the inflammatory phase, neutrophils, mast cells, and macrophages are recruited to the local defect and secrete inflammatory cytokines. During the repair stage, fibroblasts are activated by various cytokines, such as fibroblast growth factor, insulin-like growth factor, and platelet-derived growth factor which results in production of scar tissue. Multiple remodeling and degradative proteases are produced during the remodeling phase, such as MMP-1, MMP-3, and MMP-13 [5,6,8]. Although we did not quantify the levels of these inflammatory mediators in our study, it is likely that this increase in synovial fluid volume correlated to size of rotator cuff tear could represent a similar pathologic process.

Partial-thickness tears had a significantly lower volume of synovial fluid volume compared to full-thickness tears (0.76 vs 3.64 mL, respectively). We have found in our practice that frequently in the case of a questionable partial-thickness tear or “high-grade partial thickness tear” on the MRI, it is unclear how large the defect is in reality, especially when the patient fails to improve with conservative measures. During surgery, typically the tear appears much bigger than originally anticipated in our experience. This may be due to a “miss” of a full-thickness tear on the initial evaluation of the MRI by both the surgeon and the radiologist. The results of our study suggest that preoperative aspiration of the shoulder may help aid in the diagnosis of a larger clinical tear. Further investigation is required to ascertain the safety, efficacy, and mode of aspiration to best diagnose rotator cuff tears in the clinical setting.

In an attempt to determine useful clinical cutoff values for aspiration volume, we calculated the sensitivity, specificity, PPV, and NPV of aspiration volume cutoffs. An aspiration volume of 1.5 mL resulted in 91.3% specificity and 96.8% PPV for a full-thickness tear. The accuracy of this test increased to 100% specificity and PPV when the cutoff was raised to 2.5 mL. Therefore, a cutoff value greater than 1.5 mL results in high accuracy of diagnosing a full-thickness tear but increasing this cutoff to 2.5 mL or greater gives 100% likelihood of correctly identifying a full-thickness tear. However, this guideline is limited in by our patient population that were patients brought to the operative room for rotator cuff tears. 

Multiple regression analysis showed a significant correlation of smoking, tear size, and involvement of the infraspinatus to synovial fluid volume. Nicotine has been shown in multiple studies to affect rotator cuff healing in animals [11,12]. Nicotine is also believed to cause advancing arthritis; however, this remains a topic of debate [13]. The exact mechanism for the association between smoking and increased synovial fluid volume in patients with rotator cuff tears is unclear; however, this was associated in our study.

Other studies have included patients with rotator cuff tear arthropathy, including advanced degenerative changes. It is our opinion that increased inflammatory markers may have been elevated in these studies due to the presence of arthritic changes in the glenohumeral joint. Our study excluded all patients with arthroscopic evidence of arthritis, which we believe provides a more reliable association. We also excluded patients with inflammatory arthritis, and those undergoing revision surgery or those with active infection.

## Conclusions

This simple diagnostic tool could prove to be a safe, effective, and powerful adjunct for diagnosis of shoulder pathology in the clinic where MRI is not yet attained or attainable. Additionally, in the setting of surgery one should carefully evaluate the rotator cuff for a full-thickness tear if significant synovial fluid (>1.5 mL) is acquired. Future studies will focus on analyzing the synovial fluid profile and levels in patients with a variety of shoulder ailments, including labral tears and osteoarthritis. Eventually, this may provide another predictive tool for diagnosing tear size to use as an adjunct preoperative diagnostic tool.
